# 1-[(3,5-Dimethyl-1*H*-pyrazol-1-yl)carbon­yl]-5-methyl­indolizine-3-carbo­nitrile

**DOI:** 10.1107/S1600536812046727

**Published:** 2012-11-24

**Authors:** Wei-Jin Gu, Wen-Li Xie, Ting-Ting Wang

**Affiliations:** a Department of Applied Chemistry, Nanjing Normal University, Nanjing 210097, People’s Republic of China; b Key Laboratory of Applied Photochemistry, Nanjing Normal University, Nanjing 210097, People’s Republic of China

## Abstract

In the title mol­ecule, C_16_H_14_N_4_O, the indolizine ring system is essentially planar, with a maximum deviation of 0.013 (3) Å, and forms a dihedral angle of 7.52 (12)° with the pyrazole ring. In the crystal, weak C—H⋯O hydrogen bonds and π–π stacking inter­actions, with a centroid–centroid distance of 3.6378 (16) Å, link mol­ecules along [001].

## Related literature

For biological applications of indolizines and pyrazoles, see: Tukulula *et al.* (2010 ▶); James *et al.* (2008 ▶); Teklu *et al.* (2005 ▶); McDonald *et al.* (2006 ▶); Jagerovic *et al.* (2002 ▶). For background and the synthesis of related hetrocycles, see: Gu *et al.* (2011 ▶); Shen *et al.* (2006 ▶, 2008 ▶); Wang, *et al.* (2000 ▶).
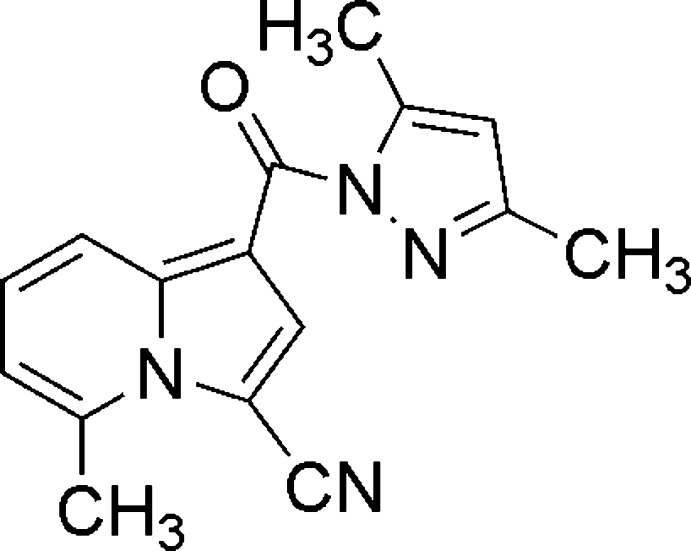


## Experimental

### 

#### Crystal data

C_16_H_14_N_4_O
*M* *_r_* = 278.31
Monoclinic, 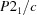

*a* = 8.5911 (18) Å
*b* = 23.3760 (15) Å
*c* = 7.5816 (12) Å
β = 114.775 (3)°
*V* = 1382.4 (4) Å^3^
*Z* = 4
Mo *K*α radiation
μ = 0.09 mm^−1^
*T* = 291 K
0.30 × 0.25 × 0.20 mm

#### Data collection

Bruker SMART APEX diffractometer
Absorption correction: multi-scan (*SADABS*; Bruker, 2000 ▶) *T*_min_ = 0.974, *T*_max_ = 0.983
7822 measured reflections
2361 independent reflections
1728 reflections with *I* > 2σ(*I*)
*R*_int_ = 0.037

#### Refinement

*R*[*F* ^2^ > 2σ(*F* ^2^)] = 0.048
*wR*(*F* ^2^) = 0.135
*S* = 1.05
2361 reflections
194 parameters
1 restraint
H-atom parameters constrained
Δρ_max_ = 0.19 e Å^−3^
Δρ_min_ = −0.15 e Å^−3^

### 

Data collection: *SMART* (Bruker, 2000 ▶); cell refinement: *SAINT* (Bruker, 2000 ▶); data reduction: *SAINT*; program(s) used to solve structure: *SHELXTL* (Sheldrick, 2008 ▶); program(s) used to refine structure: *SHELXTL*; molecular graphics: *SHELXTL* and *PLATON* (Spek, 2009 ▶); software used to prepare material for publication: *SHELXTL*.

## Supplementary Material

Crystal structure: contains datablock(s) global, I. DOI: 10.1107/S1600536812046727/lh5554sup1.cif

Structure factors: contains datablock(s) I. DOI: 10.1107/S1600536812046727/lh5554Isup2.hkl

Supplementary material file. DOI: 10.1107/S1600536812046727/lh5554Isup3.cml

Additional supplementary materials:  crystallographic information; 3D view; checkCIF report

## Figures and Tables

**Table 1:** Hydrogen-bond geometry (Å, °)

*D*—H⋯*A*	*D*—H	H⋯*A*	*D*⋯*A*	*D*—H⋯*A*
C10—H10*B*⋯O1^i^	0.96	2.56	3.435 (4)	151

Symmetry code: (i) 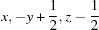.

**Fig. 1.**
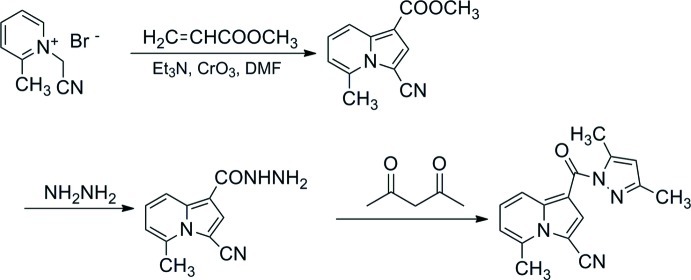
The reaction scheme.

**Fig. 2.**
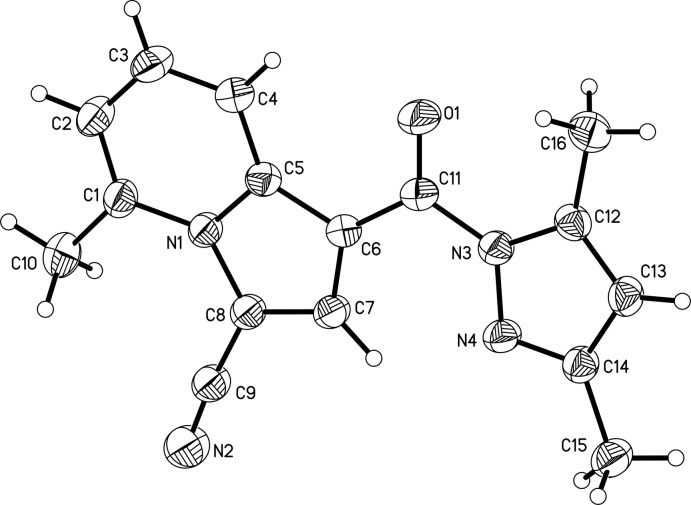
The molecular structure of title compound. Displacement ellipsoids at the 50% probability level.

**Fig. 3.**
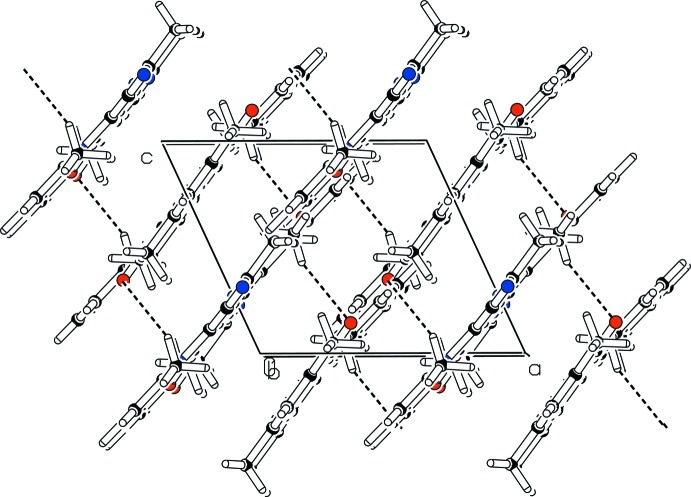
Part of the crystal structure with weak hydrogen bonds shown as dashed lines.

## supplementary crystallographic information

### Comment

Indolizines and pyrazoles are important classes of bio-active drug targets in the pharmaceutical industry, as they are the core structure of numerous biologically active compounds (Tukulula *et al.*, 2010; James *et al.*, 2008; Teklu *et al.*, 2005; McDonald *et al.*, 2006; Jagerovic *et al.*, 2002). In our continuing studies on the synthesis and properties of heterocycles (Gu *et al.*, 2011; Shen *et al.*, 2008; Shen *et al.*, 2006; Wang, *et al.*, 2000) we have prepared (Fig. 1) and determined the crystal structure of the title compound.

The molecular structure of the title compound is shown in Fig. 2. The indolizine ring system is essentially planar with a maximum deviation for atom C3 of 0.013 (3)Å. The dihedral angle formed by the indolizine ring system and the pyrazole ring is 7.52 (12)°. In the crystal, weak C—H···O hydrogen bonds and π–π stacking interactions, with Cg1···Cg2^i^ = 3.6378 (16)Å, link molecules along [001] (Fig. 3). Cg1 and Cg2 are the centroids of the N1/C5-C8 and N1/C1-C5 rings (symmetry code (i): x, 1/2-y, -1/2+z).

### Experimental

Methyl-3-cyano-5-methylindolizine-1-carboxylate was prepared through 1,3-dipolar cycloaddition according to a procedure described in the literature (Wang, *et al.*, 2000). A suspension of N-cyano-2-methylpyridinium bromide (C_5_H_4_N^+^CH_3_CN.Br^-^) (10 mmol), ethyl acrylate (40 mmol), Et_3_N (20 ml) and CrO_3_ (20 mmol) in DMF (40 ml) was stirred at 363K for 4 h (monitored by TLC). The mixture was cooled to room temperature and poured into 5% aqueous HCl (150 mL). The brown powder was collected by filtration and washed with ethanol (25 mL). After drying the solid was collected 1.28 g (60%).

Methyl-3-cyano-5-methylindolizine-1-carboxylate (5 mmol) was dissolved in 6 ml of ethanol and 20 ml 80% N_2_H_4_.H_2_O (30 mmol) was added dropwise. The solution was refluxed for 6 h and cooled to yield the product, 0.87 g (81%) as 3-cyano-5-methylindolizine-1-carbohydrazide.

3-cyano-5-methylindolizine-1-carbohydrazide (1 mmol) was dissolved in 2 ml acetic acid, then acetylacetone (2 mmol, dissolved in 2ml ethanol) was added. After stirring for 2 h, the mixture was purified by chromatography [silica gel, 20% ethyl acetate in petroleum ether (60 C90)] to yield colorless block crystals of the title compound, 0.22 g (79%). 1*H*-NMR (CDCl_3_, 400 MHz): 2.33 (s, 3H, –CH3), 2.65 (s, 3H, –CH3), 3.06 (s, 3H, –CH3), 6.06 (s, 1H, pyrazole =CH), 6.80 (d, 1H, indolizine =CH), 7.33 (t, 1H, indolizine =CH), 8.52 (d, 1H, indolizine =CH), 8.59 (s, 1H, indolizine =CH).

### Refinement

H atoms were placed in calculated positions and allowed to ride, with C—H = 0.93 and 0.96Å and *U*_iso_(H) = 1.2U_eq_(C) or 1.2U_eq_(C_methyl_). The distance for C8—C9 was restrained with the command DFIX 1.4 0.01 C8 C9 in SHELXL (Sheldrick, 2008).

### Crystal data

**Table:**

C_16_H_14_N_4_O	*F*(000) = 584
*M**_r_* = 278.31	*D*_x_ = 1.337 Mg m^−^^3^
Monoclinic, *P*2_1_/*c*	Mo *K*α radiation, λ = 0.71073 Å
Hall symbol: -P 2ybc	Cell parameters from 1864 reflections
*a* = 8.5911 (18) Å	θ = 2.1–23.1°
*b* = 23.3760 (15) Å	µ = 0.09 mm^−^^1^
*c* = 7.5816 (12) Å	*T* = 291 K
β = 114.775 (3)°	Block, colorless
*V* = 1382.4 (4) Å^3^	0.30 × 0.25 × 0.20 mm
*Z* = 4	

### Data collection

**Table:**

Bruker SMART APEX diffractometer	2361 independent reflections
Radiation source: sealed tube	1728 reflections with *I* > 2σ(*I*)
Graphite monochromator	*R*_int_ = 0.037
φ and ω scans	θ_max_ = 25.0°, θ_min_ = 1.7°
Absorption correction: multi-scan (*SADABS*; Bruker, 2000)	*h* = −10→10
*T*_min_ = 0.974, *T*_max_ = 0.983	*k* = −27→27
7822 measured reflections	*l* = −8→7

### Refinement

**Table:**

Refinement on *F*^2^	Secondary atom site location: difference Fourier map
Least-squares matrix: full	Hydrogen site location: inferred from neighbouring sites
*R*[*F*^2^ > 2σ(*F*^2^)] = 0.048	H-atom parameters constrained
*wR*(*F*^2^) = 0.135	*w* = 1/[σ^2^(*F*_o_^2^) + (0.0667*P*)^2^ + 0.2279*P*] where *P* = (*F*_o_^2^ + 2*F*_c_^2^)/3
*S* = 1.05	(Δ/σ)_max_ < 0.001
2361 reflections	Δρ_max_ = 0.19 e Å^−^^3^
194 parameters	Δρ_min_ = −0.15 e Å^−^^3^
1 restraint	Extinction correction: *SHELXTL* (Sheldrick, 2008), Fc^*^=kFc[1+0.001xFc^2^λ^3^/sin(2θ)]^-1/4^
Primary atom site location: structure-invariant direct methods	Extinction coefficient: none

### Special details

**Table:**

Geometry. All e.s.d.'s (except the e.s.d. in the dihedral angle between two l.s. planes) are estimated using the full covariance matrix. The cell e.s.d.'s are taken into account individually in the estimation of e.s.d.'s in distances, angles and torsion angles; correlations between e.s.d.'s in cell parameters are only used when they are defined by crystal symmetry. An approximate (isotropic) treatment of cell e.s.d.'s is used for estimating e.s.d.'s involving l.s. planes.
Refinement. Refinement of *F*^2^ against ALL reflections. The weighted *R*-factor *wR* and goodness of fit *S* are based on *F*^2^, conventional *R*-factors *R* are based on *F*, with *F* set to zero for negative *F*^2^. The threshold expression of *F*^2^ > σ(*F*^2^) is used only for calculating *R*-factors(gt) *etc*. and is not relevant to the choice of reflections for refinement. *R*-factors based on *F*^2^ are statistically about twice as large as those based on *F*, and *R*- factors based on ALL data will be even larger.

### Fractional atomic coordinates and isotropic or equivalent isotropic displacement parameters (Å^2^)

**Table:**

	*x*	*y*	*z*	*U*_iso_*/*U*_eq_	
C1	0.4078 (3)	0.16105 (9)	0.6276 (3)	0.0539 (6)	
C2	0.5673 (3)	0.16955 (11)	0.7688 (3)	0.0674 (7)	
H2	0.6342	0.1380	0.8292	0.081*	
C3	0.6350 (3)	0.22472 (11)	0.8269 (4)	0.0706 (7)	
H3	0.7458	0.2291	0.9227	0.085*	
C4	0.5393 (3)	0.27142 (10)	0.7437 (3)	0.0610 (6)	
H4	0.5835	0.3079	0.7834	0.073*	
C5	0.3740 (3)	0.26454 (9)	0.5980 (3)	0.0494 (5)	
C6	0.2428 (2)	0.30329 (9)	0.4823 (3)	0.0473 (5)	
C7	0.1052 (3)	0.27046 (9)	0.3576 (3)	0.0502 (5)	
H7	0.0023	0.2849	0.2655	0.060*	
C8	0.1454 (2)	0.21350 (7)	0.3920 (3)	0.0495 (5)	
C9	0.0408 (3)	0.16786 (8)	0.2894 (3)	0.0572 (6)	
C10	0.3329 (3)	0.10310 (10)	0.5630 (4)	0.0698 (7)	
H10A	0.4101	0.0746	0.6440	0.105*	
H10B	0.3150	0.0971	0.4305	0.105*	
H10C	0.2253	0.1003	0.5727	0.105*	
C11	0.2680 (3)	0.36492 (10)	0.5125 (3)	0.0553 (6)	
C12	0.1506 (3)	0.46295 (9)	0.3983 (3)	0.0514 (5)	
C13	0.0074 (3)	0.48077 (9)	0.2476 (3)	0.0565 (6)	
H13	−0.0256	0.5186	0.2140	0.068*	
C14	−0.0831 (3)	0.43187 (9)	0.1502 (3)	0.0524 (5)	
C15	−0.2500 (3)	0.42892 (10)	−0.0234 (4)	0.0673 (7)	
H15A	−0.2309	0.4329	−0.1389	0.101*	
H15B	−0.3232	0.4592	−0.0178	0.101*	
H15C	−0.3035	0.3927	−0.0256	0.101*	
C16	0.2889 (3)	0.49760 (11)	0.5473 (4)	0.0703 (7)	
H16A	0.2630	0.5375	0.5222	0.105*	
H16B	0.3963	0.4894	0.5418	0.105*	
H16C	0.2966	0.4882	0.6740	0.105*	
N1	0.3117 (2)	0.20945 (7)	0.5412 (2)	0.0481 (5)	
N2	−0.0540 (3)	0.13371 (9)	0.1932 (3)	0.0792 (7)	
N3	0.1439 (2)	0.40334 (7)	0.3880 (3)	0.0505 (5)	
N4	−0.0015 (2)	0.38486 (7)	0.2341 (3)	0.0543 (5)	
O1	0.3936 (2)	0.38496 (7)	0.6446 (3)	0.0837 (6)	

### Atomic displacement parameters (Å^2^)

**Table:**

	*U*^11^	*U*^22^	*U*^33^	*U*^12^	*U*^13^	*U*^23^
C1	0.0572 (14)	0.0536 (13)	0.0514 (13)	0.0122 (10)	0.0232 (12)	0.0077 (10)
C2	0.0593 (15)	0.0719 (17)	0.0605 (16)	0.0190 (12)	0.0147 (14)	0.0098 (12)
C3	0.0499 (14)	0.0874 (19)	0.0578 (16)	0.0108 (13)	0.0061 (12)	0.0002 (13)
C4	0.0555 (14)	0.0631 (14)	0.0525 (14)	0.0036 (11)	0.0108 (12)	−0.0049 (11)
C5	0.0492 (13)	0.0542 (13)	0.0434 (13)	0.0025 (10)	0.0179 (11)	−0.0012 (9)
C6	0.0466 (12)	0.0495 (12)	0.0415 (12)	0.0015 (9)	0.0141 (10)	−0.0008 (9)
C7	0.0452 (12)	0.0535 (13)	0.0452 (12)	0.0017 (9)	0.0125 (10)	0.0023 (10)
C8	0.0473 (12)	0.0498 (13)	0.0480 (13)	0.0019 (9)	0.0165 (11)	0.0023 (9)
C9	0.0535 (14)	0.0513 (13)	0.0578 (15)	−0.0016 (11)	0.0145 (12)	0.0037 (11)
C10	0.0733 (16)	0.0571 (15)	0.0756 (18)	0.0125 (12)	0.0280 (14)	0.0122 (12)
C11	0.0493 (13)	0.0569 (14)	0.0483 (13)	−0.0009 (10)	0.0092 (12)	−0.0052 (10)
C12	0.0494 (13)	0.0480 (12)	0.0565 (13)	−0.0065 (10)	0.0220 (11)	−0.0069 (10)
C13	0.0534 (13)	0.0433 (12)	0.0677 (15)	0.0006 (10)	0.0205 (12)	0.0015 (10)
C14	0.0503 (12)	0.0483 (12)	0.0548 (14)	0.0005 (10)	0.0183 (11)	0.0023 (10)
C15	0.0541 (14)	0.0627 (15)	0.0688 (16)	0.0000 (11)	0.0096 (13)	0.0040 (12)
C16	0.0640 (15)	0.0585 (15)	0.0795 (18)	−0.0122 (11)	0.0213 (14)	−0.0131 (12)
N1	0.0475 (10)	0.0517 (10)	0.0441 (11)	0.0068 (8)	0.0181 (9)	0.0030 (8)
N2	0.0789 (15)	0.0564 (13)	0.0822 (16)	−0.0097 (11)	0.0141 (13)	0.0027 (11)
N3	0.0496 (10)	0.0445 (10)	0.0497 (11)	−0.0015 (8)	0.0134 (9)	−0.0016 (8)
N4	0.0490 (10)	0.0487 (10)	0.0510 (11)	−0.0034 (8)	0.0071 (9)	−0.0007 (8)
O1	0.0668 (11)	0.0636 (10)	0.0771 (12)	−0.0006 (9)	−0.0126 (10)	−0.0156 (9)

### Geometric parameters (Å, º)

**Table:**

C1—C2	1.354 (3)	C10—H10B	0.9600
C1—N1	1.392 (3)	C10—H10C	0.9600
C1—C10	1.492 (3)	C11—O1	1.217 (3)
C2—C3	1.407 (3)	C11—N3	1.411 (3)
C2—H2	0.9300	C12—C13	1.347 (3)
C3—C4	1.353 (3)	C12—N3	1.395 (3)
C3—H3	0.9300	C12—C16	1.490 (3)
C4—C5	1.396 (3)	C13—C14	1.405 (3)
C4—H4	0.9300	C13—H13	0.9300
C5—N1	1.392 (3)	C14—N4	1.315 (3)
C5—C6	1.426 (3)	C14—C15	1.487 (3)
C6—C7	1.394 (3)	C15—H15A	0.9600
C6—C11	1.460 (3)	C15—H15B	0.9600
C7—C8	1.373 (3)	C15—H15C	0.9600
C7—H7	0.9300	C16—H16A	0.9600
C8—C9	1.4017 (10)	C16—H16B	0.9600
C8—N1	1.405 (3)	C16—H16C	0.9600
C9—N2	1.156 (2)	N3—N4	1.374 (2)
C10—H10A	0.9600		
			
C2—C1—N1	117.2 (2)	O1—C11—N3	117.8 (2)
C2—C1—C10	123.2 (2)	O1—C11—C6	122.1 (2)
N1—C1—C10	119.6 (2)	N3—C11—C6	120.15 (19)
C1—C2—C3	122.0 (2)	C13—C12—N3	105.07 (18)
C1—C2—H2	119.0	C13—C12—C16	129.0 (2)
C3—C2—H2	119.0	N3—C12—C16	125.9 (2)
C4—C3—C2	120.2 (2)	C12—C13—C14	107.56 (19)
C4—C3—H3	119.9	C12—C13—H13	126.2
C2—C3—H3	119.9	C14—C13—H13	126.2
C3—C4—C5	119.6 (2)	N4—C14—C13	111.09 (19)
C3—C4—H4	120.2	N4—C14—C15	120.68 (19)
C5—C4—H4	120.2	C13—C14—C15	128.2 (2)
N1—C5—C4	118.9 (2)	C14—C15—H15A	109.5
N1—C5—C6	107.16 (18)	C14—C15—H15B	109.5
C4—C5—C6	133.9 (2)	H15A—C15—H15B	109.5
C7—C6—C5	107.14 (19)	C14—C15—H15C	109.5
C7—C6—C11	132.6 (2)	H15A—C15—H15C	109.5
C5—C6—C11	120.22 (19)	H15B—C15—H15C	109.5
C8—C7—C6	109.30 (19)	C12—C16—H16A	109.5
C8—C7—H7	125.3	C12—C16—H16B	109.5
C6—C7—H7	125.3	H16A—C16—H16B	109.5
C7—C8—C9	125.51 (19)	C12—C16—H16C	109.5
C7—C8—N1	107.97 (16)	H16A—C16—H16C	109.5
C9—C8—N1	126.47 (18)	H16B—C16—H16C	109.5
N2—C9—C8	174.1 (2)	C1—N1—C5	122.08 (18)
C1—C10—H10A	109.5	C1—N1—C8	129.49 (17)
C1—C10—H10B	109.5	C5—N1—C8	108.43 (16)
H10A—C10—H10B	109.5	N4—N3—C12	111.28 (17)
C1—C10—H10C	109.5	N4—N3—C11	122.09 (16)
H10A—C10—H10C	109.5	C12—N3—C11	126.62 (18)
H10B—C10—H10C	109.5	C14—N4—N3	105.00 (16)
			
N1—C1—C2—C3	−0.1 (3)	C10—C1—N1—C5	178.91 (19)
C10—C1—C2—C3	179.9 (2)	C2—C1—N1—C8	179.34 (19)
C1—C2—C3—C4	1.1 (4)	C10—C1—N1—C8	−0.7 (3)
C2—C3—C4—C5	−0.9 (4)	C4—C5—N1—C1	1.2 (3)
C3—C4—C5—N1	−0.2 (3)	C6—C5—N1—C1	−179.24 (16)
C3—C4—C5—C6	−179.6 (2)	C4—C5—N1—C8	−179.07 (17)
N1—C5—C6—C7	−0.5 (2)	C6—C5—N1—C8	0.5 (2)
C4—C5—C6—C7	178.9 (2)	C7—C8—N1—C1	179.42 (18)
N1—C5—C6—C11	178.04 (17)	C9—C8—N1—C1	−3.2 (3)
C4—C5—C6—C11	−2.5 (3)	C7—C8—N1—C5	−0.2 (2)
C5—C6—C7—C8	0.4 (2)	C9—C8—N1—C5	177.15 (19)
C11—C6—C7—C8	−177.9 (2)	C13—C12—N3—N4	−0.3 (2)
C6—C7—C8—C9	−177.50 (19)	C16—C12—N3—N4	179.45 (18)
C6—C7—C8—N1	−0.1 (2)	C13—C12—N3—C11	178.35 (18)
C7—C6—C11—O1	172.3 (2)	C16—C12—N3—C11	−1.9 (3)
C5—C6—C11—O1	−5.8 (3)	O1—C11—N3—N4	179.5 (2)
C7—C6—C11—N3	−6.9 (3)	C6—C11—N3—N4	−1.2 (3)
C5—C6—C11—N3	175.01 (17)	O1—C11—N3—C12	1.0 (3)
N3—C12—C13—C14	0.3 (2)	C6—C11—N3—C12	−179.75 (18)
C16—C12—C13—C14	−179.5 (2)	C13—C14—N4—N3	0.0 (2)
C12—C13—C14—N4	−0.2 (3)	C15—C14—N4—N3	−179.36 (18)
C12—C13—C14—C15	179.1 (2)	C12—N3—N4—C14	0.2 (2)
C2—C1—N1—C5	−1.0 (3)	C11—N3—N4—C14	−178.54 (18)

### Hydrogen-bond geometry (Å, º)

**Table:**

*D*—H···*A*	*D*—H	H···*A*	*D*···*A*	*D*—H···*A*
C10—H10*B*···O1^i^	0.96	2.56	3.435 (4)	151

Symmetry code: (i) *x*, −*y*+1/2, *z*−1/2.

## References

[bb1] Bruker (2000). *SMART*, *SAINT* and *SADABS* Bruker AXS Inc., Madison, Wisconsin, USA.

[bb2] Gu, W.-J., Zhuang, J., Jiang, Y.-L. & Wang, B.-X. (2011). *Acta Cryst.* E**67**, o123. 10.1107/S1600536810050919 PMC3050233 21522634

[bb3] Jagerovic, N., Cano, C., Elguero, J. & Goya, P. (2002). *Bioorg. Med. Chem.* **10**, 817–827. 10.1016/s0968-0896(01)00345-5 11814871

[bb4] James, D. A., Koya, K., Li, H., Liang, G. Q., Xia, Z. Q., Ying, W. W., Wu, Y. M. & Sun, L. J. (2008). *Bioorg. Med. Chem. Lett.* **18**, 1784–1787. 10.1016/j.bmcl.2008.02.029 18308566

[bb5] McDonald, E., Workman, P. & Jones, K. (2006). *Curr. Top. Med. Chem.* **6**, 1091–1107. 10.2174/156802606777812004 16842148

[bb6] Sheldrick, G. M. (2008). *Acta Cryst.* A**64**, 112–122. 10.1107/S0108767307043930 18156677

[bb7] Shen, Y.-M., Wang, B.-X., Feng, Y.-Y., Shen, Z.-Y., Shen, J., Li, C. & Hu, H.-W. (2006). *Chem. J. Chin. Univ.* **27**, 651–653.

[bb8] Shen, Z.-Y., Wang, B.-X., Shen, J. & Hu, H.-W. (2008). *Chem. J. Chin. Univ.* **29**, 916–918.

[bb9] Spek, A. L. (2009). *Acta Cryst.* D**65**, 148–155. 10.1107/S090744490804362X PMC2631630 19171970

[bb10] Teklu, S., Gundersen, L. L., Larsen, T., Malterud, K. E. & Rise, F. (2005). *Bioorg. Med. Chem.* **13**, 3127–3139. 10.1016/j.bmc.2005.02.056 15809148

[bb11] Tukulula, M., Klein, R. & Kaye, P. T. (2010). *Synth. Commun.* **40**, 2018–2028.

[bb12] Wang, B.-X., Hu, J.-X., Zhang, X.-C., Hu, Y.-F. & Hu, H.-W. (2000). *J. Heterocycl. Chem.* **37**, 1533-1537.

